# Pomegranate Fruit as a Rich Source of Biologically Active Compounds

**DOI:** 10.1155/2014/686921

**Published:** 2014-04-10

**Authors:** Sreeja Sreekumar, Hima Sithul, Parvathy Muraleedharan, Juberiya Mohammed Azeez, Sreeja Sreeharshan

**Affiliations:** Cancer Research Program, Rajiv Gandhi Centre for Biotechnology, Thycaud P.O, Thiruvananthapuram, Kerala 695 014, India

## Abstract

Pomegranate is a widely used plant having medicinal properties. In this review, we have mainly focused on the already published data from our laboratory pertaining to the effect of methanol extract of pericarp of pomegranate (PME) and have compared it with other relevant literatures on *Punica*. Earlier, we had shown its antiproliferative effect using human breast (MCF-7, MDA MB-231), and endometrial (HEC-1A), cervical (SiHa, HeLa), and ovarian (SKOV3) cancer cell lines, and normal breast fibroblasts (MCF-10A) at concentration of 20–320 **μ**g/mL. The expressions of selected estrogen responsive genes (PR, pS2, and C-Myc) were downregulated by PME. Unlike estradiol, PME did not increase the uterine weight and proliferation in bilaterally ovariectomized Swiss-Albino mice models and its cardioprotective effects were comparable to that of 17**β**-estradiol. We had further assessed the protective role of PME on skeletal system, using MC3T3-E1 cells. The results indicated that PME (80 **μ**g/mL) significantly increased ALP (Alkaline Phosphatase) activity, supporting its suggested role in modulating osteoblastic cell differentiation. The antiosteoporotic potential of PME was also evaluated in ovariectomized (OVX) rodent model. The results from our studies and from various other studies support the fact that pomegranate fruit is indeed a source of biologically active compounds.

## 1. Introduction

*Punica granatum *L. (Punicaceae) is a nutrient dense fruit rich in phytochemical compounds [1]. Plants produce low molecular weight compounds which are broadly called phytochemicals, usually as a mechanism of defence. Some plants contain distinct families of phytocompounds, which are structurally similar to steroid hormone, 17*β*-estradiol (E2) and compete with the endogenous hormone for binding to estrogen receptor (ER), thus reducing the hormonal effect of endogenous estrogens [2–4]. These compounds are termed as phytoestrogens. Most of these phytoestrogens present in the diet are inactive compounds, which, on consumption, go through series of enzymatic changes in the gastrointestinal tract, resulting in the formation of compounds having structure similar to that of estrogens [5]. Phytoestrogens have captured major research and clinical attention due to its effectiveness in the prevention and treatment of perimenopausal and menopausal symptoms, over hormone replacement therapy (HRT) [6]. They may act both as agonists and/or antagonists in a site-specific manner, similar to the hormonal action of selective estrogen receptor modulators (SERMs) [7–9]. It can also function as antioxidants and protect DNA from oxidant-induced damage [10]. Research on pomegranate is gaining momentum due to its tremendous nutritional values and medicinal uses. The current review focuses on the use of pomegranate as a phytoestrogen rich and nutraceutical fruit with emphasis to the work done in our laboratory using methanolic extract of pericarp of pomegranate (PME).

## 2. Chemical Constituents of Pomegranate Fruit and Tree

The chemical composition of the fruits differs depending on the cultivar, growing region, maturity, cultivation practice, climate, and storage circumstances [11]. About 50% of the total fruit weight corresponds to the peel, which is an important source of bioactive compounds such as phenolics, flavonoids, ellagitannins, and proanthocyanidin compounds, minerals, mainly potassium, nitrogen, calcium, phosphorus, magnesium, and sodium, and complex polysaccharides. The edible part of the pomegranate fruit (50%) consists of 40% arils and 10% seeds. Arils contain 85% water, 10% total sugars, mainly fructose and glucose, and 1.5% pectin, organic acid, such as ascorbic acid, citric acid, and malic acid, and bioactive compounds such as phenolics and flavonoids, principally anthocyanins [12]. The seed cover of the fruit contains delphinidin-3-glucoside, cyanidin-3-glucoside, delphinidin-3,5-diglucoside, cyanidin-3,5-diglucoside, pelargonidin-3,5-diglucoside, and pelargonidin-3-glucoside with delphinidin-3,5-diglucoside being the main anthocyanin in pomegranate juice [13]. 12–20% of total seed weight of pomegranate comprises seed oil and is self-possessed with more than 70% of the conjugated linolenic acids. The fatty acid component of pomegranate seed oil comprises over 95% of the oil, of which 99% is triacylglycerols. Minor components of the oil include sterols, steroids, and a key component of mammalian myelin sheaths, cerebroside [14, 15]. Interestingly, punicic acid, which is a conjugated isomer unique to pomegranate oil, constitutes 70–76% of the seed oil [16]. Phenolic compounds, together with flavonoids, anthocyanins, and tannins, are the main group of antioxidant phytochemicals that are important due to their biological and free radical scavenging activities [17]. Phenolic acids, flavonoids, and tannins are present in different parts of pomegranate fruit and this may be one of the reasons why many of the studies demonstrated that combinations of pomegranate extracts from different parts of the fruit were more effective than a single extract [18]. In a comparative analysis, anthocyanins from pomegranate fruit were found to possess higher antioxidant activity than vitamin-E (*α*-tocopherol), *β*-carotene, and ascorbic acid [19].  Table 1 represents the key constituents of pomegranate fruit and tree [20–41].

## 3. Therapeutic Functions of Pomegranate

**Figure 1:**
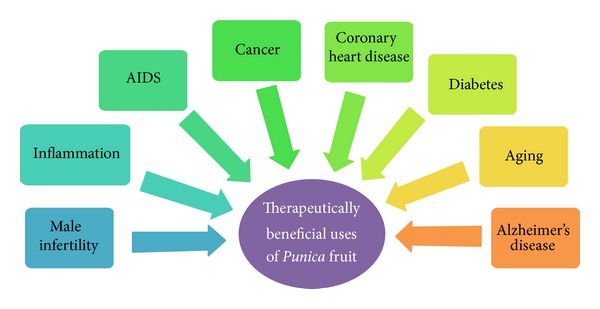
Therapeutically beneficial uses of* Punica* fruit. Pomegranate fruit has been proven to act against various diseases like cancer, cardiovascular disorders, diabetes, AIDS, and Alzheimer's disease.

Extracts of all parts of the pomegranate fruit exhibit therapeutic properties [15] and target a range of diseases including cancer, cardiovascular disorders, diabetes, male infertility, Alzheimer's disease [42], aging, and AIDS [43] (Figure 1). Although pomegranate's extensive therapeutic benefits may be attributed to a number of mechanisms, most researchers have determined its antioxidant, anticarcinogenic, and anti-inflammatory properties. Various therapeutic applications of* Punica granatum* are discussed here.

### 3.1. Cancer

Research on breast cancer cell lines demonstrated that pomegranate constituents efficiently inhibited angiogenesis [44], invasiveness [40], growth [45], and induced apoptosis [46]. Its anti-invasive, antiproliferative, and antimetastatic effects were attributed to the modulation of Bcl-2 proteins, upregulation of p27 and p21, and downregulation of cyclin-cdk network [47]. Pomegranate constituents inhibit angiogenesis via downregulation of vascular endothelial growth factor (VEGF) in human umbilical vein endothelial and MCF-7 breast cancer cell lines [44], thereby hampering the tumor growth. Prostate cancer cells, when treated with pomegranate juice, increased adhesion and decreased the migration. Molecular analyses revealed that pomegranate juice increased the expression of cell-adhesion related genes and inhibited the expression of genes involved in cytoskeletal function and cellular migration. It would possibly affect prostate cancer because of its apoptotic, antioxidant, antiproliferative, and anti-inflammatory properties, suggesting that it may be beneficial in slowing down or preventing cancer cell metastasis [48]. The application of pomegranate extract to the skin of mice before they were exposed to a carcinogenic agent was shown to inhibit the appearance of erythemas and hyperplasia and the activity of epithelial ornithine decarboxylase [49]. An* in vivo* study in TRAMP mice model suggested that oral supplementation of pomegranate fruit extract inhibited metastasis and increased overall survival [50].

**Figure 2:**
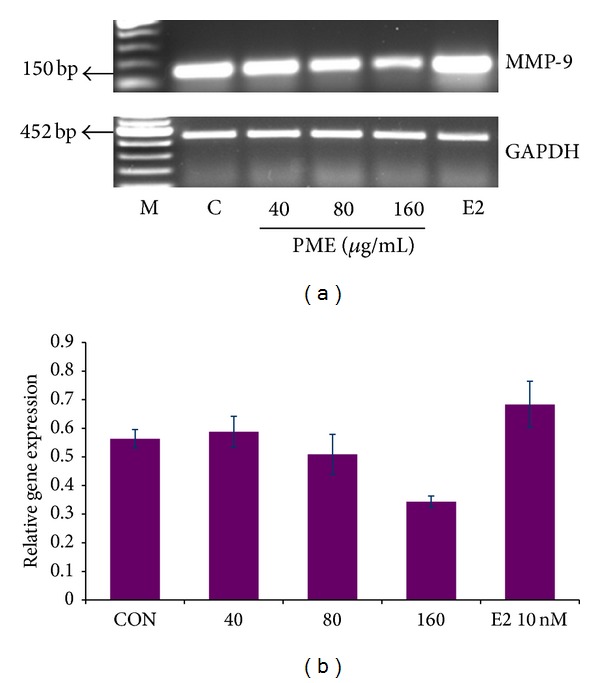
Effect of PME on MMP-9 transcription in MCF7 cells. (a) MCF7 cells were incubated with PME (40, 80, and 160 *μ*g/mL) and E2 (10 nM) for 24 hrs and semiquantitative RT-PCR was done. (b) shows the ratio of density of MMP-9 gene expression to that of endogenous control GAPDH and it represents mean ± SE of 3 replicates (*P < 0.05).

Matrix metalloproteinases (MMPs) are good markers of tumor cell invasion and migration [51]. Phytochemicals have been shown to target the activity and secretion of MMPs in estrogen responsive cancers [52]. Constituents of pomegranate minimize tumor cell invasion into normal tissue and metastasis to distant sites and these actions develop due to the inhibition of selected metalloproteinase activity, decreased focal adhesion kinase activity, and reduced VEGF expression [15]. With semiquantitative RT-PCR, we had found out that PME downregulated the transcription of MMP-9 suggesting its possible role in the inhibition of tumor invasion (Figure 2) whereas E2 (10 nM) did not significantly affect the transcription of MMP-9 [53] which correlated with earlier studies suggesting that estrogen stimulated MMP-9 secretion without increasing its gene transcription [54].

**Figure 3:**
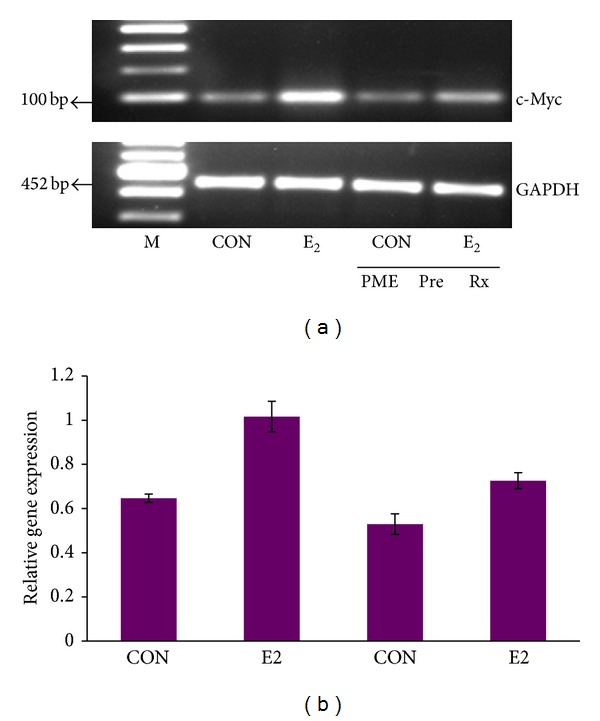
Effect of PME pretreatment on E2 induced expression of c-Myc. (a) MCF7 cells were pretreated with 100 nM E2 for 4 hrs, with or without PME pretreatment (80 *μ*g/mL) for 12 hrs and RT-PCR was done. (b) shows the ratio of density of c-Myc gene expression to that of endogenous control GAPDH and it represents mean ± SE of 3 replicates (*P < 0.05).

**Figure 4:**
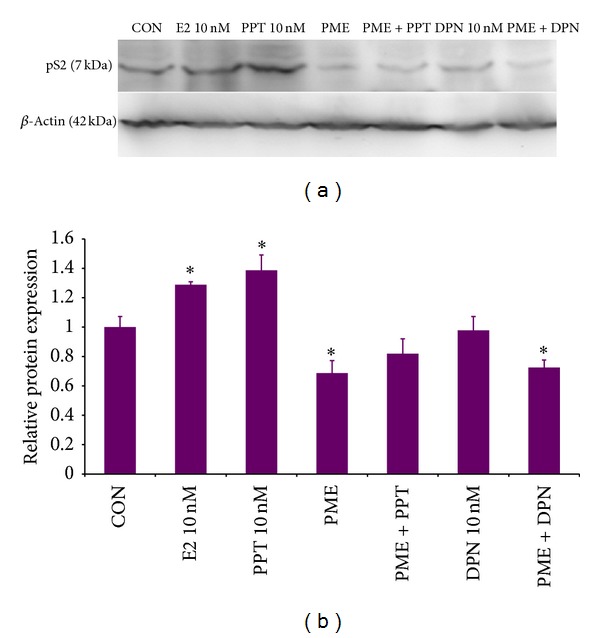
Effect of PME on pS2 protein expression in MCF7 cells. (a) Immunoblot result of MCF7 cells treated with 10 nM E2, PME (80 *μ*g/mL), 10 nM PPT, 10 nM DPN, and PME (80 *μ*g/mL) with 10 nM PPT or 10 nM DPN for 48 hrs. (b) Data are presented as densitometric ratio relative to nontreated control (CON) cells. The values are mean ± SE of 3 separate experiments (*P < 0.05).

We had assessed the estrogenicity/antiestrogenicity of PME in a panel of* in vitro* biological assays and the expression of endogenous estrogen sensitive markers (pS2 and PR) in breast carcinoma cell lines were analyzed [53]. When MCF-7 cells pretreated with PME were treated with estrogen, the c-Myc expression was not induced as much as when treated with estrogen alone, demonstrating the effect of PME in estrogen regulated mechanism (Figure 3). ER positive cells treated with PPT (4,4′,4′′-(4-Propyl-(1*H*)-pyrazole-1,3,5-triyl)*tris*phenol) (ER*α* selective agonist) and DPN (Diarylpropionitrile) (ER*β* selective agonist) clearly showed that PPT increased the pS2 protein levels, whereas DPN did not produce any significant effect. When given in combination with PPT, PME reduced the pS2 protein levels indicating the role of ER*α* in mediating the effects of PME on pS2 expression (Figure 4). Thus the effect of PME on expression of pS2 was mediated by ER*α* and not by ER*β* [53].

Pomegranate fruit extract was revealed to inhibit UV-B-mediated phosphorylation of mitogen-activated protein kinase (MAPK) and nuclear factor NF-*κ*B activation [55]. Pomegranate juice almost downregulated the TNF*α* induced Akt (protein kinase B) activation required for NF-*κ*B activity [56]. Koyama et al. [57] examined the effects of pomegranate extract (POMx) on the IGF system and found out cell growth inhibition and apoptosis. Their findings suggested that POMx treatment reduced mTOR phosphorylation at Ser2448 and Ser2481, whereas IGFBP-3 increased phosphorylation at those sites. These results suggested that POMx decreased prostate cancer cell survival by inhibiting IGF1 expression. To conclude, pomegranate fruit has anticancer properties that can be attributed to different mechanisms.

### 3.2. Cardiovascular Disorders

*In vitro*,* in vivo* and human trials had examined the effects of a range of pomegranate constituents on the prevention and reduction of atherosclerosis and LDL oxidation [58]. Evidence suggested that polyphenolic antioxidants contained in pomegranate juice can cause reduction of oxidative stress and atherogenesis through the activation of redox-sensitive genes ELK-1 and p-JUN and increased eNOS expression. Their results indicated that proatherogenic effects induced by disturbed shear stress can be reversed by constant administration of pomegranate juice [59]. Pomegranate juice consumption for 3 years by patients with carotid artery stenosis reduced common blood pressure, LDL oxidation, and carotid intima-media thickness [60]. Azadzoi et al. demonstrated that 8-week administration of pomegranate juice concentrate daily in a rabbit model of arteriogenic erectile dysfunction significantly increased intracavernous blood flow and smooth muscle relaxation, probably via its antioxidant effect on enhanced nitric oxide preservation and bioavailability [61]. A pilot study in type 2 diabetic patients with hyperlipidemia found that concentrated pomegranate juice decreased cholesterol absorption, increased faecal excretion of cholesterol, had a favourable effect on enzymes concerned in cholesterol metabolism, drastically reduced LDL cholesterol, and improved LDL/HDL cholesterol and total/HDL ratios [62]. Aviram et al. analyzed atherosclerotic lesion size, antioxidant activity, blood sugar, peritoneal macrophages, oxidative status, and lipid profiles for 3 months after giving 6 different pomegranate preparations with varying amounts of total polyphenols and gallic acid content in atherosclerotic apolipoprotein-E deficient mice and found that pomegranate phenolics and pomegranate unique complexed sugars could mimic the antiatherogenic effects of pomegranate extracts [63]. All these evidences suggest the potential cardioprotective effect of pomegranate fruit.

### 3.3. Antiosteoporotic Potential

**Figure 5:**
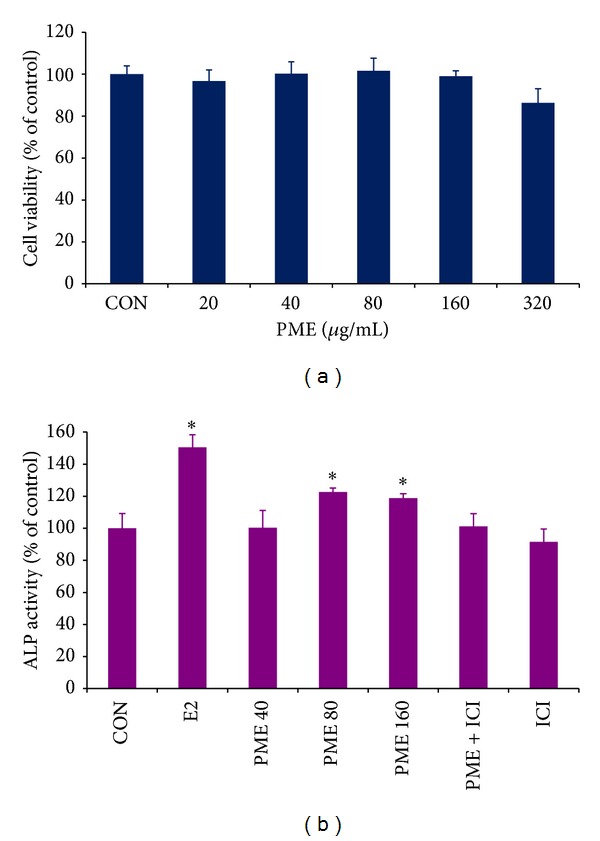
PME increased ALP activity in MC3T3-E1 osteoblast without affecting cell proliferation. (a) Dose-dependent effect of PME on cell proliferation. MC3T3-E1 osteoblasts were treated with 0 (control), 20, 40, 80, 160, and 320 *μ*g/mL of PME for 48 hrs and the cell proliferation was determined by MTT assay. The cell proliferation was expressed as percentage cell viability over the untreated control. (b) Cells were treated with E2 (10 nM), PME (40, 80, and 160 *μ*g/mL), ICI (1 *μ*M) with or without PME (80 *μ*g/mL) for 48 hrs. ALP activity was then measured and results are expressed as percentage over the untreated control. Results are expressed as mean values ± SE of five replicates. *P < 0.05 when compared to untreated control.

Tissue selective estrogen agonist/antagonists are currently being investigated as alternatives to estrogen in the prevention and treatment of postmenopausal osteoporosis [64–66]. Bone loss after ovariectomy is associated with high bone turnover where bone resorption rate exceeds the bone formation rate [67]. To assess the protective role of* Punica* on skeletal system, we had examined the effect of PME on a well-characterized osteoblastic cell population (osteoblastic MC3T3-E1 cells) and examined its effect on Alkaline Phosphatase (ALP), which is a commonly used bone remodelling marker. The results (Figure 5) indicated that PME significantly increased ALP activity, supporting its suggested role in modulating osteoblastic cell differentiation [68].

**Figure 6:**
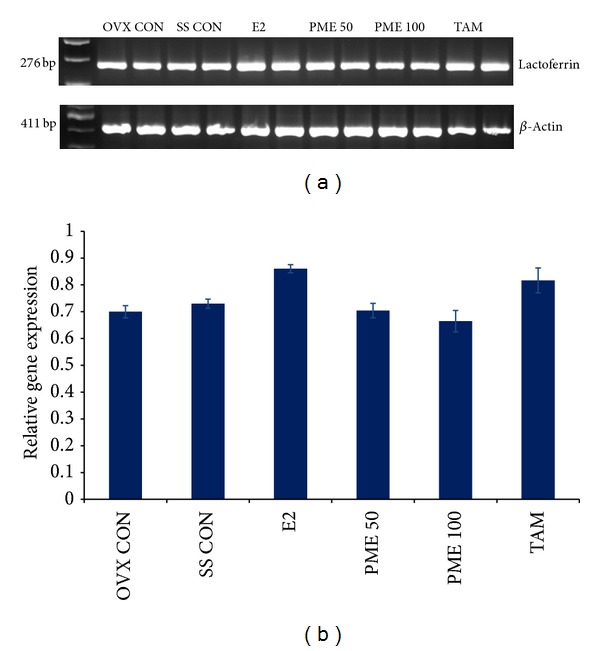
PME did not alter lactoferrin expression in murine uterus. (a) RT-PCR detection of Lactoferrin m-RNA in sham control (SS CON) and ovariectomized mice exposed to 0.1% ethanol (vehicle control, Ovx CON), E2 (1 mg/kg bwt), PME (50, 100 mg/kg bwt), and tamoxifen (TAM, 10 mg/kg bwt) for 7 days. (b) shows the ratio of density of target gene expression to that of endogenous control beta-actin and it represents mean ± SE of three replicates.

Ovariectomized rodent model is a well-established system for estrogen deficiency induced bone loss and used by researchers previously [69, 70]. We had evaluated the antiosteoporotic potential of the extract in estrogen deficiency induced osteoporosis in young adult mice of 6–8 weeks of age by assessing the bone turnover by serum ALP. In comparison to the sham surgery (SS) control, ovariectomized (Ovx) control animals showed an increase in ALP activity indicating an increase in bone turn-over rate in these animals. PME in higher concentration was found to be effective in decreasing this bone turnover, though E2 was better in controlling the accelerated bone turnover (Table 2). The experimental model differed from aged Ovx mice wherein the osteoporosis is induced only by estrogen deficiency and not by a combination of natural bone loss due to age and ovarian hormone deficiency. An increase in bone turn-over rate was indicated by higher serum ALP level in the Ovx group compared to the SS control group. Therefore, high rate of bone turnover was well corrected by PME suggesting that it might play a protective role against ovarian hormone insufficiency related bone resorption. But E2 as well as PME was able to significantly decrease ALP levels in Ovx mice (Table 2). Serum calcium and phosphorous levels in Ovx control, PME treated, and tamoxifen treated animals were similar to that of SS control animals. Significant decrease in calcium levels was observed in E2 treated animals in comparison to SS control (Table 2). Our findings clearly indicated that the possible bone preserving effect of PME is almost comparable with E2 [53]. Earlier studies had shown that an acute or chronic exposure to xenoestrogens or dietary phytoestrogens alters uterine expression of estrogen sensitive genes in mice [71]. So in order to check whether PME has any effect, a semiquantitative RT-PCR was done to analyze uterine mRNA levels of lactoferrin in ovariectomized mice fed with PME for 7 days. Lactoferrin is a well-known estrogen target gene and a biologically active molecule for bone regeneration [72]. The positive control E2 increased the uterine accumulation of lactoferrin mRNA in Ovx animals compared to the vehicle treated Ovx control (Figure 6). Lactoferrin expression did not differ significantly between the groups that received PME (50, 100 mg/kg bwt) and the vehicle (0.1% ethanol) treated Ovx control group, indicating the lack of estrogenicity of PME on uterine endometrium in the doses tested in our study. Tamoxifen (10 mg/kg bwt) was found to increase the expression of lactoferrin, though not significantly [68]. As there are promising results from both* in vitro* and* in vivo* studies, we suggest evaluating the antiosteoporotic potential by clinical trials with pomegranate fruit extract that has no side effects on uterine endometrium alongside a significant decrease in bone turn-over rate.

### 3.4. Other Clinical Applications

*In vitro *assay showed that fermented pomegranate juice extract is better than red wine and comparable to green tea [37]. There were also reports that pomegranate juice possessed considerably greater antioxidant capacity at much lower concentrations (>1000-fold dilutions) than either grape or blueberry juice [73].* Punica granatum *peel extract decreased lipid peroxidation in hepatic, cardiac, and renal tissues and at the same time it had a facilitatory effect on the scavenging capability of superoxide anion and hydrogen peroxide [74]. Formerly, it was shown that pomegranate peel extract supplementation alleviated oxidative damage of the liver and enhanced the hepatic structure and function in rats exposed to bile duct ligation [75]. Pretreatment of carbon tetrachloride-induced liver damage in rats with pomegranate peel extract resulted in the reduction of lipid peroxidation and at the same time, the free-radical scavenging activity of catalase, superoxide dismutase, and peroxidase were considerably enhanced [76]. Many studies had keenly explored the anti-inflammatory properties of pomegranate fruit [15, 77–79]. Studies indicated that pomegranate extract inhibited PMACI-induced proinflammatory cytokines assembly by inhibiting the gene expression. This is achieved by blocking JNK and ERK-MAPK activation and NF-*κ*B activation in human KU812 cells [80]. Larrosa et al. showed that pomegranate extract supplementations led to reduced prostaglandin E2 (PGE2) levels in the colon mucosa by downregulating the overexpressed COX-2 and prostaglandin E synthase (PTGES) levels owing to the action of ellagic acid [78].* Punica granatum *extract had been found to be particularly effective for controlling oral inflammation, dental plaque, and bacterial and fungal counts in periodontal disease and Candida-associated denture stomatitis [81, 82]. Another study proposed that inhibition of number of signal transduction pathways and the downstream pathogenic cellular response by pomegranate extract or compounds may be a useful approach for the prevention of the onset and severity of inflammatory arthritis [77]. The dynamism of pomegranate fruit in newer areas of pharmacological effects might be delivered in the future.

## 4. Pomegranate Extract as a Phytoestrogen

Due to the possible adverse side effects of estrogenic stimulation (such as increase in tumor risk), many women have turned to phytoestrogens as an alternative for HRT [83]. The features that facilitate the chemicals to bind with ER are the steric and hydrophobic properties of a compound, as well as the hydrogen bonding between the phenolic hydroxyl group and the ER binding site [84]. Phytoestrogens bind to both forms of ER and showed a lower binding affinity than E2. Some of them exhibit a higher binding affinity to ER*β* than to ER*α* which may indicate that they have different pathways for their actions and explains tissue specific changeability in phytoestrogenic action [85]. Both genomic and nongenomic mechanisms have been projected to explain phytoestrogenic effects on human health [86]. The best move towards the avoidance and handling of estrogen-dependent breast cancer is to selectively hold estrogen activity in the affected tissues without compromising its beneficial effects [87]. Regrettably, at this time, available antiestrogen such as tamoxifen used in the treatment of ER-positive breast cancer has side effects and agonism in the uterine endometrium, leading to an uncertain connection to endometrial carcinoma [88–90]. A competitive radioactive binding study was done to ascertain whether PME interacts with ER and had shown that PME binds to ER and inhibited the binding of labelled estrogen to ER in a dose-dependent manner [53, 91].

## 5. Pomegranate as a Potential Nutraceutical

According to De Felice, who coined the term nutraceutical, it can be defined as, “a food (or part of a food) that provides medical or health benefits, including the prevention and/or treatment of a disease” [92]. It may range from isolated nutrients, herbal products, dietary supplements, and diets to genetically engineered “designer” foods and processed products such as cereals, soups, and beverages [93, 94]. Anthocyanidins (delphinidin, cyanidin, and pelargonidin) and hydrolysable tannins (such as punicalagin, pedunculagin, punicalin, gallagic, and ellagic acid esters of glucose), account for the major antioxidant activity of whole fruit [22, 95]. The peel, which is also a major part of the fruit, is an imperative source of bioactive compounds such as phenolics, flavonoids, ellagitannins, proanthocyanidin compounds [96], minerals, [97], and complex polysaccharides [98]. Aviram and others reported that systolic blood pressure was reduced, after 1 year of pomegranate juice consumption. This was believed to be related to the potent antioxidant properties of pomegranate polyphenols [60]. Hong et al. confirmed that pomegranate juice and pomegranate extracts were more potent inhibitors of cell growth than isolated individual polyphenols in cell lines, influential synergistic and/or additive effects of several phytochemicals including proanthocyanidins, anthocyanins, and flavonoid glycosides [99]. Pomegranate contains agents, particularly polyphenolic flavonoids, which exert actions that could be well conducive to good oral health, particularly in relation to gingivitis development [100]. Pomegranate juice had the greatest antioxidant potency composite index among beverages like black cherry juice, cranberry juice, grape juice, apple juice, orange juice, red wines, blueberry juice, and iced tea; and the antioxidant activity was at least 20% superior to any of the other beverages tested [101–103]. Each and every part of pomegranate provides health benefits, that is, a nutraceutical food.

## 6. Summary and Conclusions

**Figure 7:**
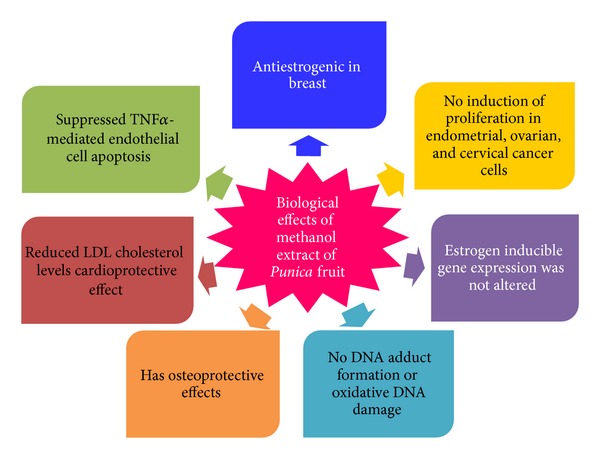
Biological effects of PME. PME was found to be antiestrogenic in breast, exhibited cardioprotective and osteoprotective effects, and had no estrogenicity in uterus. It did not induce DNA adduct formation or oxidative DNA damage and suppressed TNF *α*-mediated endothelial cell apoptosis.

The discovery that plants generate hormonally active phytochemicals has altered our understanding of the connection between diet and human health. It is well established that fruit or plant extracts are a complex mixture of various constituents and, in most of the instances, it is not clear whether a single compound or a mixture of compounds is responsible for the reported effects [104]. The thought of the whole herb or multiherb preparation not only addresses multiple targets, but possibly will alleviate the toxicity and side effects of a single, isolated compound from the plant. Many* in vitro* and* in vivo* studies pointed out high nutritional and potential tissue specific action of extract of* Punica granatum*. Proofs are accumulating that compounds present in a fruit or herb extract augment each other's biological effect. For example, it has been reported that quercetin and ellagic acid (both are also present in pomegranate) together make use of a more prominent inhibitory effect against cancer cell growth than either compound alone [105]. We had found that PME has antiestrogenic effect in the mammary gland, without compromising the beneficial effects of estrogen in the cardiovascular and skeletal system and had no estrogenicity in the uterus [53]. PME could possibly be considered as an ideal SERM and further studies might demonstrate its suitability and possible application in estrogen dependent breast cancers with beneficial effects in other hormone dependent tissues.  Figure 7 describes the biological effects of PME, as observed in our studies. Furthermore, it would be valuable to investigate the long-term effects of PME in the* in vivo* models of estrogen deprivation to demonstrate its suitability in HRT. To achieve this goal, a better understanding is needed regarding the orchestrated action of SERM, receptor and coregulators that contribute to distinct patterns of gene expression. Although scientific research is being carried out to study the biological activity of a lot of food phytochemicals, the health claims attributed to the final marketed nutraceutical products have normally little or doubtful scientific foundation. This is owing to the fact that a great deal of scientific conclusion is derived from animal testing and* in vitro* assays, while human clinical trials are limited. Some key issues such as metabolism, bioavailability, toxicity, and dose/response of these food bioactive compounds or nutraceuticals themselves have not been well recognized yet. Currently, numerous clinical trials are in progress exploring the therapeutic potential of pomegranate extracts. Its potential use as a nutraceutical needs to be investigated. We may thus anticipate that many of the open issues about the biological effect of extract of* Punica granatum* will be answered in the near future.

## Figures and Tables

**Table 1:** Principal constituents of different parts of pomegranate tree and fruit. The different parts of pomegranate plant like peel, root, bark, flower, leaves, and so forth exhibit different phytochemicals.

Pomegranate peel	Pomegranate juice	Pomegranate root and bark	Pomegranate flower	Pomegranate leaves	Pomegranate seed
(i) Gallic acid (ii) Ellagic acid (iii) Punicalin (iv) Punicalagin (v) Caffeic acid (vi) Ellagitannins (vii) Pelletierine alkaloids (viii) Luteolin (ix) Kaempferol (x) Quercetin	(i) Simple sugars (ii) Aliphatic organic acids (iii) Gallic acid (iv) Ellagic acid (v) Quinic acid (vi) Flavonols (vii) Amino acids (viii) Minerals (ix) EGCG (x) Ascorbic acid	(i) Ellagitannins (ii) Piperidine alkaloids (iii) Pyrrolidine alkaloid (iv) Pelletierine alkaloids	(i) Gallic acids (ii) Ursolic acid (iii) Triterpenoids (iv) Fatty acids	(i) Carbohydrates (ii) Reducing sugars (iii) Sterols (iv) Saponins (v) Flavanoids (vi) Tannins (vii) Piperidine alkaloids (viii) Flavone (ix) Glycoside (x) Ellagitannins	(i) 3,3′-Di-*O*-methylellagic acid (ii) 3,3′,4′-Tri-*O*-methylellagic acid (iii) Punicic acid (iv) Oleic acid (v) Palmitic acid (vi) Stearic acid (vii) Linoleic acid (viii) Sterols (ix) Tocopherols (x) Sex steroids

References [20–26]	References [15, 20, 26–30]	References [21, 23]	References [31–33]	References [21, 22, 34, 35]	References [36–41]

**Table 2:** Effect of E2, PME, and tamoxifen on chosen markers of bone metabolism of ovariectomized mice. Serum calcium, phosphorus, and Alkaline Phosphatase (ALP) levels of sham control (SS Con) and Ovx mice exposed to 0.1% ethanol (vehicle control), E2 (1 mg/kg bwt), PME (50, 100 mg/kg bwt), and tamoxifen (TAM, 10 mg/kg bwt) for 7 days (bwt = body weight). Data are expressed as mean ± SE (*n* = 5).

	Sham control	OVX control	E2 (1 mg/kg bwt)	PME (50 mg/kg bwt)	PME (100 mg/kg bwt)	TAM (10 mg/kg bwt)
Calcium (mg/dL)	9.46 ± 0.313	10.94 ± 1.18	8.188 ± 0.7040^a ^	8.5 ± 0.707	9.09 ± 0.194	9.908 ± 0.165
Phosphorus (mg/dL)	7.43 ± 0.63	8.818 ± 0.698	8.026 ± 1.066	7.516 ± 1.731	8.78 ± 1.980	8.146 ± 0.0680
ALP (U/L)	140.6 ± 11.28	181.8 ± 34.07^a ^	115.2 ± 23.61^b ^	130.4 ± 12.77	120 ± 9.02^b ^	134.6 ± 17.54^b ^

^a^ *P* < 0.05 versus sham control, ^b^ *P* < 0.05 versus ovx control.
